# Compression of the lower trunk of the brachial plexus by a cervical rib in two adolescent girls: case reports and surgical treatment

**DOI:** 10.1186/1749-7221-4-14

**Published:** 2009-09-06

**Authors:** Lars B Dahlin, Clas Backman, Henrik Düppe, Harukazu Saito, Anette Chemnitz, Kasim Abul-Kasim, Pavel Maly

**Affiliations:** 1 Hand Surgery, Department of Clinical Sciences in Malmö, Lund University, Malmö, Sweden; 2 Department of Hand Surgery, Malmö University Hospital, Malmö, Sweden; 3 Department of Hand Surgery, Norrland University Hospital, Umeå, Sweden; 4 Department of Orthopaedic Surgery, Malmö University Hospital, Malmö, Sweden; 5 Department of Orthopaedic Surgery, Murayama Medical Center, National Hospital Organization, Tokyo, Japan; 6 Department of Radiology, Malmö University Hospital, Malmö, Sweden

## Abstract

Presence of a cervical rib in children is extremely rare, particularly when symptoms of compression of the lower trunk of the brachial plexus occur. We present two cases with such a condition, where two young girls, 11 and 16 years of age were treated by resection of the cervical rib after a supraclavicular exploration of the lower trunk of the brachial plexus. The procedure led to successful results, objectively verified with tests in a work simulator, at one year follow-up.

## Background

A cervical rib, articulating into the first rib is typically an asymptomatic condition that is even discovered incidentally. Clinical symptoms from the lower trunk of the brachial plexus by the cervical rib are less frequent. In a pediatric population, a cervical rib with neurogenic symptoms is an extremely rare condition with only single cases treated and reported [1-3]. In the published case reports, resection of the first rib and the attached cervical rib has been done through an axillary or a supraclavicular approach with successful postoperative result at one month after surgery, but long-term results are not available. We present two cases with compression of the lower trunk of the brachial plexus by a cervical rib in two young girls, 11 and 16 years old. The condition was successfully treated by resection of the cervical rib through a supraclavicular approach. At one year follow-up, both patients remained free of recurrent symptoms.

### Case one

**Figure 1:**
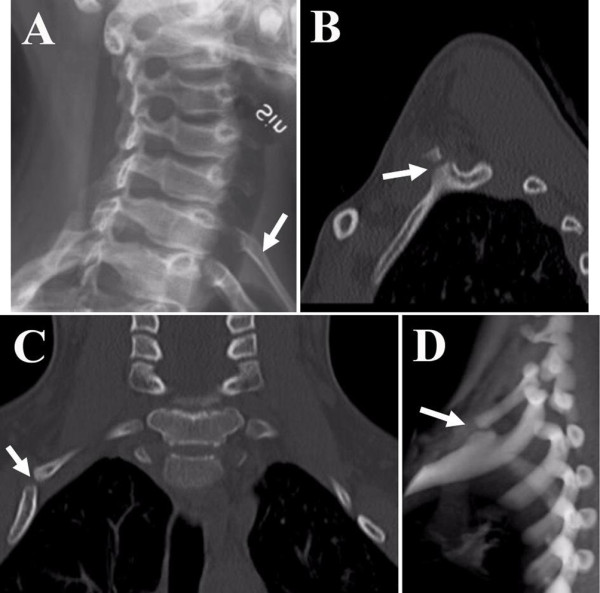
**Case 1: (A) Plain radiograph oblique view showing the right cervical rib (arrow)**. (B-D) CT sagittal, coronal and 3D-reconstructed images showing the pseudoarthrotic bony formation (arrows) between the cervical rib and the first rib.

**Figure 2:**
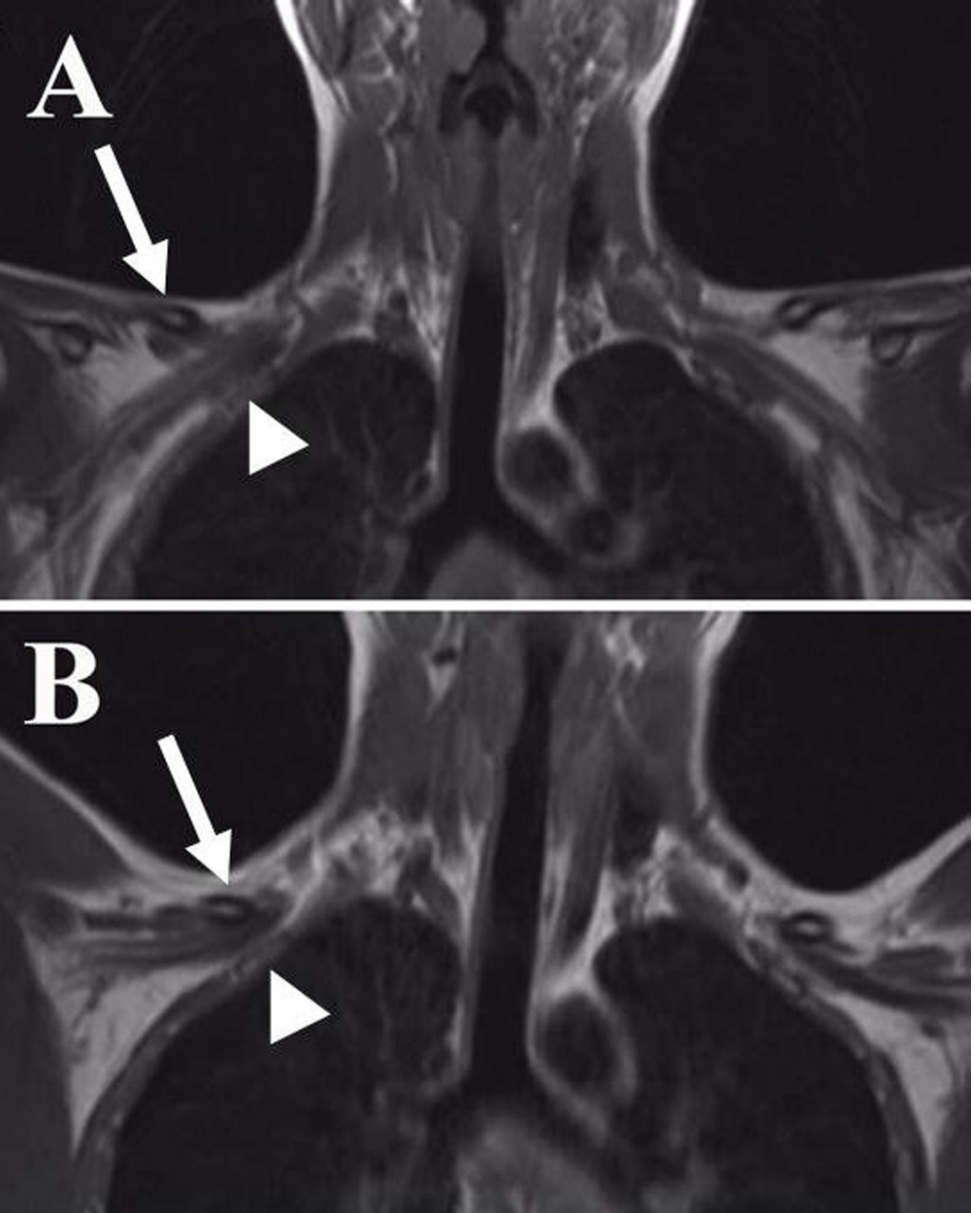
**Case 1: (A-B) MRI T1-weighted coronal images showing that the space between the cervical rib (arrow) and the first rib (arrow head) diminishes upon lifting the upper arm with subsequent impingement of the brachial plexus in image B**.

An 11 year old right-handed girl with a history of a bilateral tumour in the neck was referred to our hospital for a second opinion. She had previously been examined at another hospital due to a tumour on the right side. Diagnosis was based on a conventional X-ray and a biopsy which showed bone tissue. No further treatment was done. We had no information available of the diagnostic and treatment considerations from that hospital. The girl also had symptoms such as paraesthesia and pain in the middle ring and little fingers, particularly on the right side, often during night time. The history of the patient included fatigue and pain while writing and working on a computer. She had problems carrying things in the hands, especially when the arm was pulled in the axial direction. Lifting the arms above the shoulder plane elicited similar symptoms in the fingers on the right side. She experienced intolerance to cold. Range of motion in the shoulder, elbow, wrist and fingers was normal, but she expressed pain in the three ulnar fingers during abduction above 90 degrees. She had impaired internal rotation/adduction/extension ("hand on the back") on the right side. Examination showed palpable cervical ribs bilaterally, where percussion in the area elicited symptoms in the three ulnar fingers. Subjectively, she expressed a somewhat impaired sensibility in the little fingers, particularly on the right side. The strength of the first dorsal interosseous muscle and the other ulnar nerve innervated muscles was equal (no atrophy in the extremity) to the contralateral side, but she had a positive Froment's sign. Two-point discrimination (2-PD) was 2-3 mm in all fingers. A normal pulse in the radial artery was noted even with the arm lifted. Assisted hand assessment (AHA) showed no abnormality. Isometric and dynamic tests of the right hand in a work stimulator (BTE Primus) showed 8-10% lower values than in the left hand. Electrophysiological investigation showed no abnormalities except a slightly increased F-wave (latency 18.9 ms; upper border 18.1). No EMG recordings were done from individual intrinsic muscles of the hand. Radiographs and CT of the cervical spine showed bilateral cervical ribs articulating against a bone prominence on the cranial surface of the first rib (Fig. 1). The cervical rib with the "pseudoarthrotic" bony formation slightly dislocated the lower part of the brachial plexus ventrally. On MRI performed with the arms lifted, the space between the cervical rib, the bone formation and the clavicle decreased (Fig. 2). MRI also showed fibrous tissue formation around the pseudoarthrotic bone formation. There were no similar findings of the brachial plexus on the left side despite the presence of a cervical rib.

**Figure 3:**
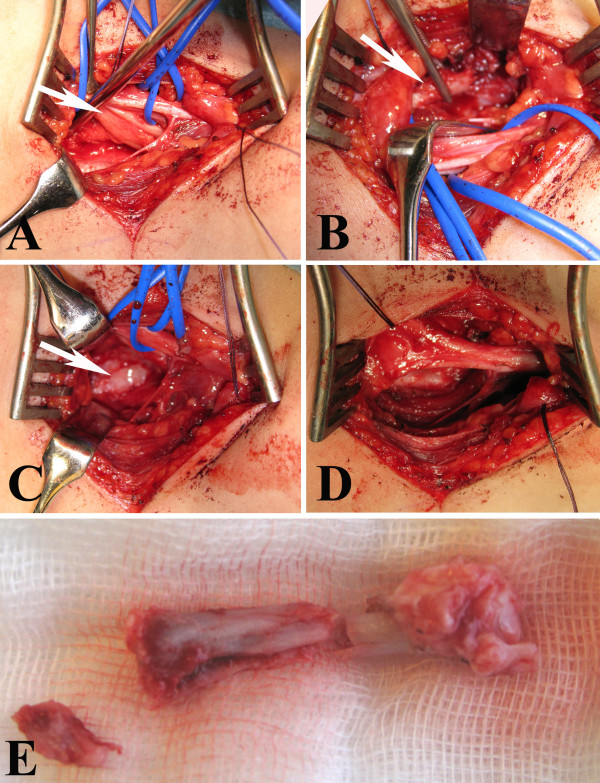
**Surgical exposure and resection of the cervical rib on the right side of the 12 year old girl (Case 1)**. The brachial plexus was explored via a supraclavicular approach (arrow lower trunk; A), revealing the cervical rib (arrow; B), which was resected. The resected bone surface was concealed with bone wax (arrow; C). After exploration, the brachial plexus, particularly the lower trunk was no longer riding above the cervical rib (D). The resected cervical rib is shown in E.

When the patient was 12 years old, the cervical rib and the brachial plexus on the right side was explored supraclavicularly. The inferior trunk was riding over the cervical rib while the subclavian artery was located ventral to the cervical rib and the bone formation (Fig. 3). The artery was not affected. The entire cervical rib including periosteum and fibrotic bands was resected. Thereafter, no anatomical structures disturbed the lower trunk. The postoperative events were uncomplicated, except initial pain during deep breath (conventional X-ray of the lungs showed no pathological findings). She was treated with the anti-inflammatory drug diclofenac to theoretically reduce new bone formation.

At regular follow-up at 1, 3, 6 and 12 months, she had no remaining symptoms from the lower trunk of the brachial plexus, except a slight allodynia around the scar during the first six months. She had no symptoms during full abduction. Cold intolerance was markedly reduced (none or insignificant) and a Froment's sign was not found. At one year follow-up, she had full range of motion and no impairment of strength compared to the contralateral side. Endurance, isometric and dynamic grip strength showed 9-18% higher values than on the left side. The girl was pleased with the surgical procedure. She continued her leisure activities in gymnastics.

### Case two

**Figure 4:**
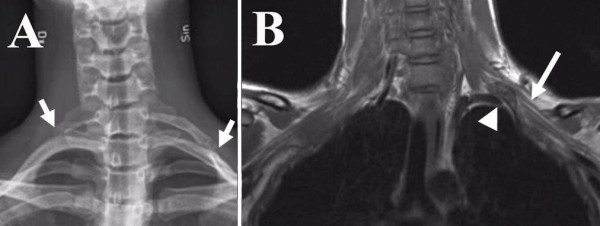
**Case 2: (A) Frontal radiograph showing bilateral cervical ribs, shorter on the right side**. The lateral end of the cervical ribs is marked with arrows. (B) MRI T1-weighted coronal image showing the cervical rib (arrow head, B) with its pseudoarthrotic bony formation that lifts up the brachial plexus (long arrow).

A 16-year old right-handed girl with paraesthesia in the left arm, initially occurring periodically and later more frequent, since the age of 12 was referred to our hospital due to these symptoms. X-ray showed a cervical rib on the left side and a minor one on the right side (no symptoms on right side; Fig. 4). She had similar symptoms as in Case One, such as paraesthesia and numbness in the three ulnar fingers of the left hand when carrying things in the hand, when a pressure was applied supraclavicularly (e.g. carrying a backpack) or when working with the hands above the plane of the shoulder. Percussion of the area of the palpable cervical rib on the left side elicited symptoms in the three ulnar fingers and "hands up tests" exaggerated the symptoms in the same fingers. The radial pulse was normal in all positions of the arm. She had good strength in all muscles of the upper extremity and a normal sensibility in the hand. Isometric test and endurance of grip showed 32% and 62%, respectively and weakness in the left hand compared to the right side (BTE Primus work simulator). Isometric test of the flexion in the left shoulder and endurance showed 16% and 54%, respectively lower values, compared to the right side. Electrophysiological examination showed no abnormalities. MRI showed a 6 cm long cervical rib from C7 on the left side, which articulated against a cranially oriented bony process from the first rib where the articulation was bulky (Fig. 4). The left brachial plexus was slightly lifted up by the skeletal abnormality. On the asymptomatic right side a 2.5 cm long cervical rib was found, which had no contact with the brachial plexus.

**Figure 5:**
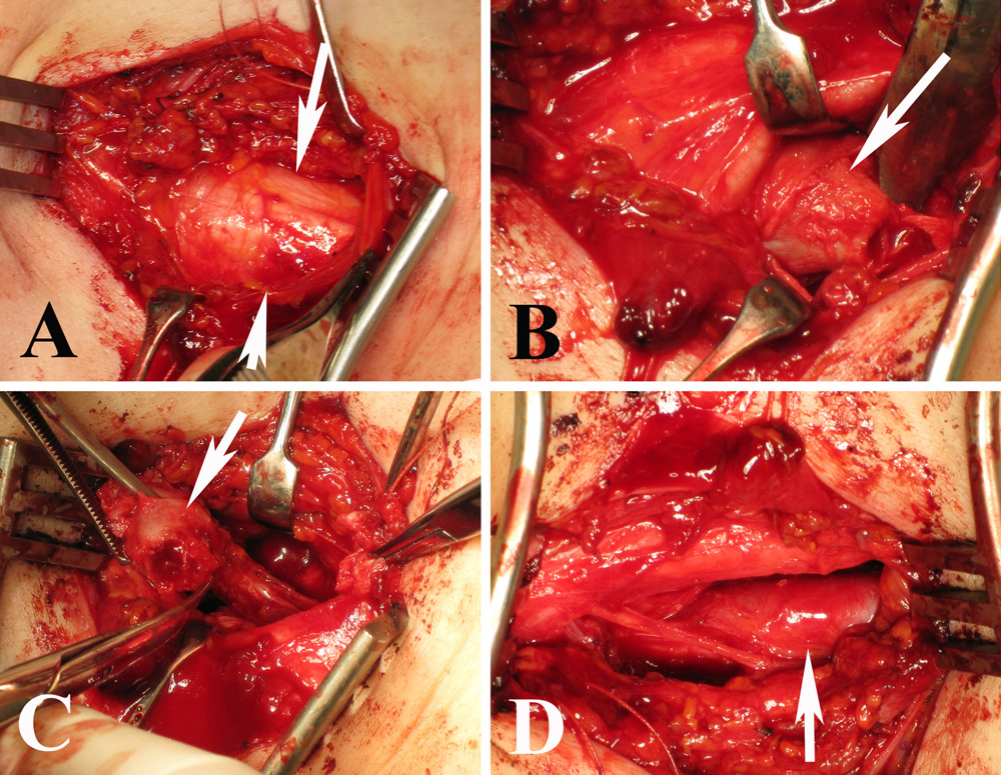
**Exploration of the brachial plexus through a supraclavicular approach on the left side of the 17 year old girl**. After skin incision and incision of the fascia the brachial plexus (arrow; A) was located very superficially riding on the cervical rib (arrow; B) and with a distorted anatomy of the brachial plexus rather twisted and horizontally located on top of the cervical rib. The cervical rib was resected in pieces (large arrow cervical rib; small arrow fibrous tissue; C) and the surface of the remaining exposed bone was covered with bone wax. After resection of a cervical rib, the brachial plexus was no longer distorted by any structures and the subclavian artery could be observed (arrow in D). Photos taken from below with the left arm to the right and the head to the left.

The brachial plexus and the cervical rib of the patient were explored when the girl was 17 years. The brachial plexus was distorted at and adhered to the ventral edge of the cervical rib and the bony process from the first rib (Fig. 5). The main part of the cervical rib including the bone process from the first rib was resected after the lower trunk was lifted up (Fig. 5). The subclavian artery was not impinged by the bone formation. The direct postoperative events were without problems, but later she was investigated at the Department of Infectious Diseases due to fever of unknown origin. No cause of the fever was found and later she recovered completely. She was followed regularly as with Case One.

At one year follow-up she had no symptoms in the hand. The preoperative symptoms had disappeared although she still experienced a feeling of impaired strength in the left arm. She had full range of motion and it was not possible to provoke any paraesthesia. Tests of fine motor activity in the hand (Crawford pins and sleeve and Minnesota picking test) showed improved values. Tests in the work simulator showed improvement [isometric test 5% weakness (preoperatively 32%), endurance 54% weaker (preoperatively 62%), isometric test of extension with elevated arm 4% weaker than the right side (preoperatively 16%), endurance of flexion/extension with elevated arm similar value on the right side (preoperative 54% weaker)]. MRI follow-up 11 months after surgery revealed no occurrence of the resected cervical rib. There were no differences compared to the two CT-scans done at three and six months after surgery (done for other reasons; fever investigation and a fall from a horse). The patient was pleased with the surgery. She continued with her previous studies and leisure activities without restriction.

## Discussion

Our patients had cervical ribs bilaterally, but mainly experienced unilateral symptoms, where resection of only the symptomatic cervical rib through a supraclavicular approach was successfully done in both cases. Both girls had symptoms and a history, including pain at night time with a clear suspicion that the lower trunk of the brachial plexus was affected since carrying heavy things and lifting the arm above the shoulder and other activities elicited paraesthesia and numbness particularly in the ulnar part of the hand. Objectively, the impaired function in the arm and the hand was clearly demonstrated with the various tests using a work simulator, indicating the usefulness of such novel investigation pre- and postoperatively in patients with compression of the brachial plexus. The symptoms of the patients corresponded to the findings in the clinical examination and the MRI, indicating the value of MRI. Preoperatively, neurography and EMG did not reveal any specific impairment of nerve function, except an increased F-wave in Case One. However, MRI showed a clear affection of the brachial plexus from the cervical rib in both cases when imaging was done with the arm abducted. This indicates that MRI should be done in the positions that elicit symptoms. The MRI findings were verified when the lower trunk of the brachial plexus was explored. In both cases the nerve structures were riding over the cervical rib with fibrous bands approaching the lower trunk.

Compression of one or more of the neurovascular structures traversing the superior aperture of the chest is generally referred as thoracic outlet syndrome (TOS). This syndrome has been the focus in a large number of articles including description of neurophysiologic examinations, surgical techniques and results, see for example [4-14]. But only a few papers have focused on children and adolescents [15], and on the importance of cervical rib for irritation of the brachial plexus and the subclavian artery [9]. A thorough history should be taken and appropriate investigations should be undertaken in patients with a suspected TOS to define the cause of symptom and exclude other diagnoses [4].

In contrast to a previous report [2], our patients did not have any muscular wasting, but only sensory symptoms, probably explaining the lack of electrophysiological alterations. In both cases, there was a successful relief of symptoms with a complete recovery in the younger girl and with just minor remaining intermittent symptoms in the older girl, at the one-year follow- up. In addition, the preoperative tests performed at our hand rehabilitation unit demonstrated a clear improvement of the results at the regular follow-up at 3, 6 and 12 months. In addition, we could objectively demonstrate improvement by examination of various tasks using the work simulator, indicating its usefulness in pre- and postoperative investigations, which has not been previously utilised. Electrophysiological criteria for neurogenic thoracic outlet syndrome have previously been suggested, such as low amplitude of the median compound muscle action potentials, low or relatively low ulnar sensory nerve action potentials, relatively low amplitude or normal ulnar compound muscle action potential and normal-amplitude median sensory nerve action potential [16]. We found that the electrophysiological investigation showed no abnormalities, which maybe due to the fact that the lower trunk was affected to a limited extent in contrast to other published cases [2]. Electrodiagnostic procedures have previously been discussed in the literature [4,9,12]. The brachial plexus in Case Two had a distorted (rotated; a horizontal rather than a vertical plane) direction caused by the cervical rib and the bony formation. We could not observe any signs that the subclavian artery was compressed between the rib and the fibrous bands even if it has been reported that a cervical rib of more than 5.5 cm long tends to lift up and kink the subclavian artery [3].

We decided to explore the lower trunk through a supraclavicular approach to be able to explore the impact of the cervical rib on the lower trunk due to the disturbing, mainly sensory, symptoms in the patients. Advantages of a supraclavicular exploration for thoracic outlet syndrome have been presented earlier with few reported complications after such approach as compared to a transaxillary resection of the first rib [9,17,18], but conflicting opinions exist about the best approach [10]. The presence of a cervical rib and fibrous band form a barrier over which particularly the lower trunk of the brachial plexus enters the arm with a potential microtrauma to the trunk by stretching and compression [19,20]. Interestingly, even if our present cases had similar cervical ribs bilaterally (just a short one on the right side in the older girl), symptoms only occurred on one side. In the contralateral side of the younger girl the symptoms were extremely rare and therefore no indication for exploration. In the older girl, symptoms occurred on the side where the cervical rib was more prominent; thus, only a rudimentary cervical rib was presented on the asymptomatic side.

## Conclusion

We suggest that the presence of a cervical rib even in children may induce true nerve compression, where the symptoms vary with position of the arm causing mainly sensory symptoms in the distribution of the lower trunk. These patients should be carefully examined and investigated, including MRI and various tests in work simulator. The possibility of surgical exploration with resection of the cervical rib should be considered in appropriate cases. We advocate a supraclavicular approach with a careful exploration of the lower trunk and resection of the cervical rib, the bony formation from the first rib and fibrous bands.

## Consent

Informed consent was obtained from the patients and their parents for publication of this case report and any accompanying images. A copy of the written consent is available for review by the Editor-in-Chief of this journal.

## Competing interests

The authors declare that they have no competing interests.

## Authors' contributions

LD, CB, HD, AC and HS operated the patients. The radiological examinations were performed by KAK and PM. All authors contributed to the creation of the manuscript and have read/approved the manuscript.
